# Seasonal and diel activity patterns of the endangered taiga bean goose (*Anser fabalis fabalis*) during the breeding season, monitored with camera traps

**DOI:** 10.1371/journal.pone.0254254

**Published:** 2021-07-15

**Authors:** Milaja Nykänen, Hannu Pöysä, Sari Hakkarainen, Tuomas Rajala, Juho Matala, Mervi Kunnasranta

**Affiliations:** 1 Department of Environmental and Biological Sciences, University of Eastern Finland, Joensuu, Finland; 2 Natural Resources, Natural Resources Institute Finland (Luke), Joensuu, Finland; 3 Natural Resources, Natural Resources Institute Finland (Luke), Helsinki, Finland; Wildlife Conservation Society Canada, CANADA

## Abstract

Taiga bean goose (*Anser fabalis fabalis*) is an endangered subspecies that breeds sporadically in remote habitats in the arctic and boreal zones. Due to its elusive behaviour, there is a paucity of knowledge on the behaviour of taiga bean goose during the breeding season, and survey methods for monitoring numbers in the breeding areas are lacking. Camera traps are a useful tool for wildlife monitoring, particularly when there is a need for non-invasive methods due to the shy nature of the species. In this study, we tested the use of camera traps to investigate seasonal and diel activity patterns of taiga bean goose in Finland over two successive breeding seasons, 2018 and 2019. We did this by modelling counts of geese from images with generalized linear and additive mixed models. The camera type (cameras placed by experts specialized in bean goose ecology *vs* randomly placed cameras) did not influence the count of taiga bean goose (p = 0.386). However, the activity varied significantly by region, Julian day, time of day and temperature, with the study site (individual peatland) and year adding substantial random variation and uncertainty in the counts. Altogether, the best fitting model explained nearly 70% of the variation in taiga bean goose activity. The peak in activity occurred about a month later in the southernmost region compared to the more northern regions, which may indicate behaviours related to migration rather than breeding and moulting. Our results show that long-term monitoring with game camera traps provide a potential unobtrusive approach for studying the behavioural patterns of taiga bean goose and can increase our ecological knowledge of this little-known subspecies. The results can be applied to planning of the annual censuses and finding the optimal time frame for their execution.

## Introduction

Finding a reliable and cost-effective method to survey and monitor animal populations is a prerequisite for successful management and conservation of wildlife [1–3]. This is particularly challenging with species that occur in low numbers and have a scattered distribution. This is the case with the bean goose, *Anser fabalis*, a species breeding sporadically in remote and inaccessible habitats in the arctic and boreal zones from Fennoscandia to Western and Eastern Siberia [4, 5]. The conservation status of bean goose is globally categorized as of Least Concern in the International Union for the Conservation of Nature Red List [6]. However, the bean goose is currently divided into three or four subspecies depending on genetic markers used [7] (but see also [5]), and the status of the subspecies *A*. *f*. *fabalis*, the taiga bean goose, is more worrying in its main breeding areas, being classified as Endangered in Northwest Russia (order of the Ministry of Natural Resources of Russian Federation #162 from 24 March 2020, Diana Solovyeva, personal communication) and Vulnerable in Finland [8] and in Sweden [9]. Based on mid-January counts in the wintering areas [10], population size of the taiga bean goose has decreased to such a degree that hunting of the species is managed within an International Single Species Action Plan under the Agreement on the Conservation of African-Eurasian Migratory Waterbirds [11].

While taxonomy and various population genetic aspects of the bean goose subspecies have been studied at some length [7, 12, 13] and basics of the migration patterns of the species are known based largely on neck-banded and satellite tagged individuals [14, 15], only sporadic information exists on the behavioural patterns of breeding individuals [16]. During the breeding season lasting from the end of April to August, taiga bean goose is elusive and even detection of birds is difficult. Indeed, one of the biggest challenges in the conservation of the declining taiga bean goose is the lack of a suitable survey method for monitoring its numbers in the breeding areas [11, 17]. Developing survey protocols for the species is challenging, because very little is known about the seasonal and diel activity patterns of the taiga bean goose during the breeding season. Such information is difficult to obtain in the field, because the detectability of taiga bean goose may be further reduced by disturbance caused by the presence of human observers [17].

During recent decades, camera trapping has become a reliable non-invasive tool for wildlife monitoring, especially for large mammals, but increasingly also for avian species. It substantially reduces survey efforts and minimizes observer interference [18, 19]. Camera trapping is particularly well suited for studying various behavioural traits of elusive species such as activity patterns [18, 20–22]. Camera traps have also been found useful for estimating abundance and densities of birds and mammals [23–26]. However, camera traps also have limitations that need to be accounted for when planning their use in monitoring. For example, the placement of camera traps may introduce bias in the data and affect inferences made about activity patterns [18, 22, 27, 28].

Here, we first wanted to assess the applicability of camera trapping per se as a method to survey taiga bean goose numbers in the breeding areas. Related to this goal, we examined if there is a difference in the data provided by randomly placed cameras and cameras that were placed by experts, both camera types being installed in the same peatlands that were potential breeding habitats. If the two camera types gave similar results, cameras placed by citizens (non-experts) could be used to gather regionally more comprehensive data for monitoring purposes.

Secondly, we explored the possibility to use camera trapping to measure seasonal and diel activity patterns of the rare and elusive taiga bean goose during the breeding season. Specifically, we wanted to gain critically needed information on the temporal activity patterns of the taiga bean goose, i.e. the timing of the breeding season and time of day the geese are most active and thus most likely to be captured by both camera traps and visual surveys. This would inform us about the appropriate timing of abundance surveys done during the late breeding season when the geese are moulting. We examined the activity patterns while accounting for a range of environmental variables because they may affect the activity of adults and goslings via changes in thermoregulatory and foraging demands [29]. In addition, temperature, precipitation and wind have often been considered environmental factors that affect the survival of offspring and breeding success of precocial species [30–32], including geese [33]. We expected that adverse weather conditions (i.e., low temperature, high precipitation and strong wind) would decrease the activity of adults and goslings.

## Materials and methods

**Fig 1:**
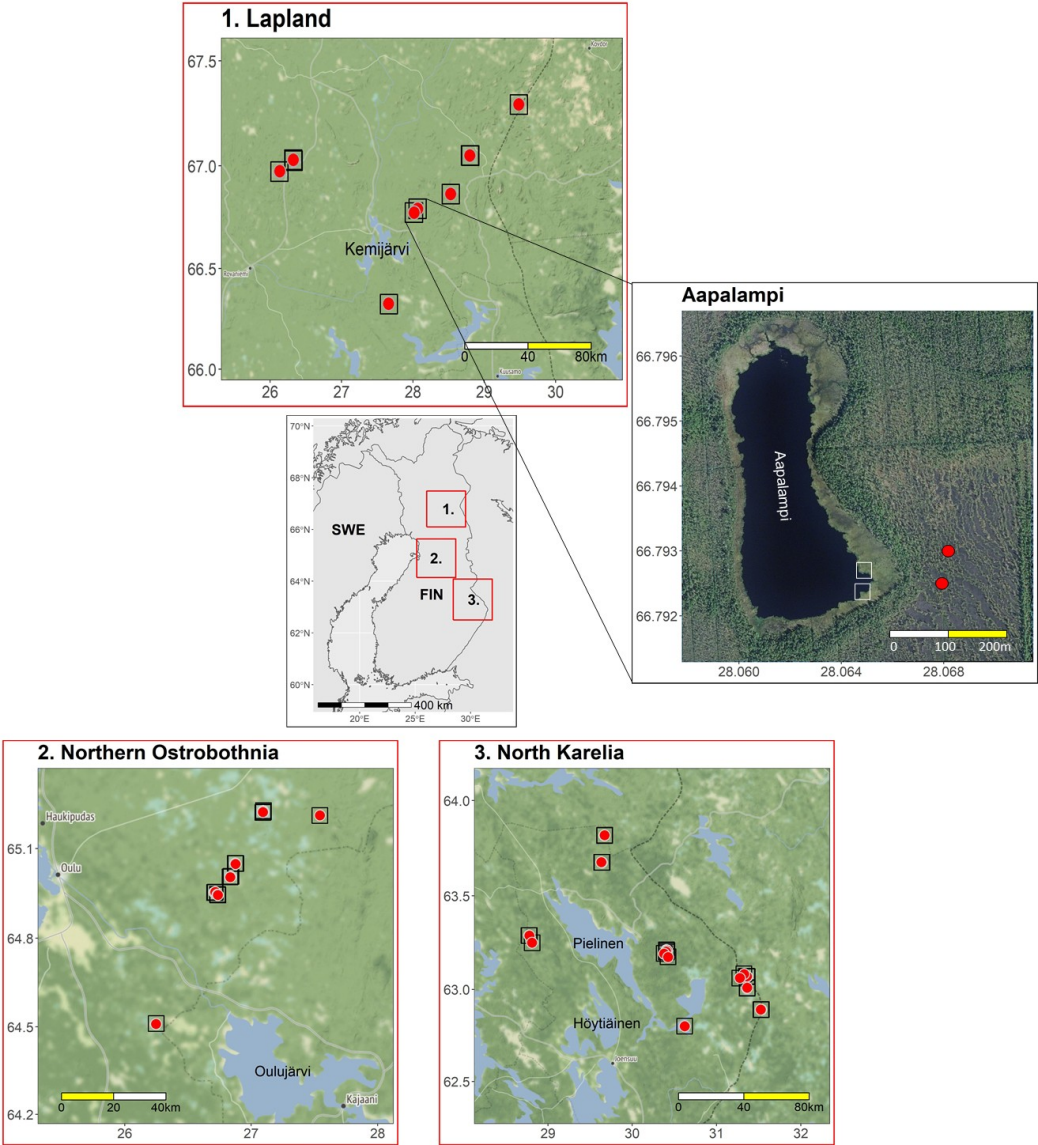
Study areas. Location of the study areas (in provinces of Lapland, Northern Ostrobothnia, and North Karelia), the peatlands where 52 and 92 camera traps were deployed in 2018 and 2019, respectively, and a close up of one peatland, Aapalampi, in Lapland province. Solid red circles are locations of cameras placed randomly and grey open squares locations of cameras placed by experts. Note that due to the map scale the camera locations overlap. The maps were drawn using R-packages *ggplot2* [34] and *ggmap* [35]. The image of Aapalampi was drawn using aerial orthophotos of the National Land Survey of Finland (CC BY 4.0), and the images of the study regions using map tiles by Stamen Design, under CC BY 3.0 (data by OpenStreetMap, under ODbL). The inset map was drawn using Natural Earth map data.

According to the international species conservation action plan, taiga bean geese in Finland belong to the central management unit which breeds in the boreal coniferous forest zone in northernmost Sweden, Northern and Central Finland, Northeast Norway, Russian Karelia, the Kola Peninsula and Arkhangelsk district [11]. The study regions covered altogether 17 and 30 known bean goose peatlands across Finland in 2018 and 2019, respectively. The peatlands were located in the provinces of North Karelia (N = 9/14), Northern Ostrobothnia (N = 6/8) and Lapland (N = 2/8) (Fig 1). Depending on the year, 52 and 92 camera traps were installed in the breeding seasons from the beginning of May to August, covering the time period when the geese were nesting, caring for the offspring and moulting. In each study location, 2–4 game camera units (Uovision UV785 Full HD 12 MP with 16 GB memory cards) were placed on peatlands with ponds/water bodies. Study locations were divided into 6.25 ha grids and one or two grids were used for camera trap monitoring, depending on size of the peatland. In each square, the first camera was randomly placed (camera type ‘random’) and the location for the second camera was chosen by experts specialized in taiga bean goose ecology (camera type ‘expert’). Both camera types were placed overlooking the shoreline of the pond, and the distance between cameras within a pair varied between 15 and 264 m, depending on the study site (see Fig 1). Cameras were attached to a tree or wooden pole at a height of 1 m above the ground. Motion sensitive cameras were set to capture two still images with a 10 s delay between triggers. Triggering distance of a camera depends on the size of the moving object with a single bean goose triggering the camera from a maximum of 10–15 meters. Cameras were typically visited once or twice during the study period to replace the memory cards and batteries, if needed. Taiga bean geese were not intentionally approached during the study and remote camera traps are considered to be a non-invasive study method. Therefore, no permits concerning animal welfare/ethics for bean goose camera trapping were required. Camera traps were set with landowners’ permission on private properties and on state-owned areas with Metsähallitus permit (permit number MH1145/2018).

After each field season, adult taiga bean geese and goslings were detected and counted manually from the images. Also other avian and mammalian species were identified, as for example the presence of competitors or predators may affect the presence of taiga bean goose. Diel patterns in taiga bean goose activity were studied by dividing 24 h into 12 two-hour periods (i.e., 00:00–01:59, 02:00–03:59,… but for simplicity we later denote them as 00–02, 02–04,…) and counting the total number of geese in each of the periods; the maximum number of geese observed per two hours was recorded in the database.

Generalized linear mixed modelling (GLMM) approach was used to investigate whether there was a difference in total taiga bean goose numbers (adults and goslings added together) captured by camera type ‘random’ compared to camera type ‘expert’. We removed the peatlands without any goose images recorded throughout the year and ran the GLMM in R [36] using the package *glmmTMB* [37] with terms ‘year’ (2018 and 2019) and ‘study site’ (altogether 26 peatlands) included as random intercepts in the model. The varying effort, resulting from the different number of cameras deployed on the study sites and the amount of time that the cameras were recording over the study period, was accounted for by including an offset term in the model that was calculated by totalling the number of time periods of recording per camera type for each day. Due to the data being zero-inflated count data (some days and time-periods had zero goose counts), we ran different candidate models with a Tweedie, zero-inflated Poisson and two types of negative Binomial distributions (nbinom1 and nbinom2). We then compared the model fit using Akaike’s Information Criterion (AIC, [38]) values to determine the best fitting model.

We used Tweedie error distribution, and generalized additive models, GAMs [39] and generalized additive mixed models, GAMMs [40, 41], to investigate the effect of a set of geographic, environmental and temporal parameters on taiga bean goose activity, here defined as the total number (or count) of adult bean geese and their goslings captured by game cameras during a two-hour period of each day. The explanatory covariates (see S1 Table) included the wider geographical study area (provinces of Lapland, Northern Ostrobothnia, and North Karelia) and study site (peatland), the latter being treated as a random variable as we were not interested in the effect of study site *per se* but wanted to quantify the variation caused by it. The temporal covariates included year (treated as random variable), Julian day (representing seasonal activity) and time period (representing diel activity), consisting of 12 equally spaced two-hour periods (*i*.*e*., 00–02, 02–04, …, 22–00) for each day. The environmental variables included the mean, minimum and maximum air temperature (°C), rain accumulation (mm) and wind speed (m/s), retrieved from the open portal of the Finnish Meteorological Institute (http://opendata.fmi.fi/wfs), and averaged for each two-hour time period, for each study site (see S2 Table for range of the variables). The models also included the offset term quantifying the varying amount of camera effort.

For the retrieval of the environmental variables, a custom R-script was used to extract the hourly data from all weather stations located within 50 km to the study sites, after which the extracted variable values (*v*) were interpolated for each site using averaging with square-exponential weighting by distance: $v=exp(\frac{{-d}^{2}}{\delta^{2}})$, where *δ* is the bandwidth of 50,000 m and *d* is the distance (in meters) from the study site to the weather station.

In the first step of modelling taiga bean goose activity, a set of GAMs were run using the R-package *mgcv* [42] with smoothness selection performed with Maximum Likelihood while excluding the random variables year and study site, and the deviance explained, significance and AIC values were compared to determine which of the environmental covariates should be carried to the random effects model (S1 Table). Due to the collinearity between the minimum, maximum and mean temperature, and the fact that these variables explain more or less the same phenomenon, we chose to include only mean temperature in the models. There was no evidence of concurvity between any of the other variables (with all concurvity estimates of <0.4), thus they were all included in the same model. After this, various random effect structures were added (*e*.*g*., year and study site as random intercepts, random slope of Julian day within each year, random smooth of Julian day within each year) and their relative fit to the data tested by running the candidate models with smoothness selection performed with Restricted Maximum Likelihood and comparing the AIC values (S1 Table). We also included interactions between province and Julian day and province and time period as we wanted to investigate whether the temporal variation in taiga bean goose activity varied between the different regions (caused for example by latitudinal difference). The interaction province*Julian day*time period was included to further investigate the possible change in goose activity with time of day and with Julian day, separately for each province. The significance of each model term was assessed based on p-values, and non-significant terms dropped. The model fits were assessed by inspecting plots of residuals and response *vs* fitted values created with *mgcv* function ‘gam.check’, and the predictions for the best fitting model were plotted at the response level, using functions ‘plot_smooth’ and ‘vis.gam’ from R-package *itsadug*, for each significant (at *ɑ* = 0.05 level) model variable and variable pair (interaction), respectively, while holding the other variables at their mean.

## Results

All in all, the cameras produced over 566,000 images, the majority of which were empty (*i*.*e*., not containing images of geese or other animals) due to false triggering caused by *e*.*g*., moving leaves. Fifteen out of the 17 study locations in 2018 and 24 out of the 30 locations in 2019 produced geese images. Taiga bean geese were present in altogether 756 out of 45,053 time periods that had camera effort (at least one camera recording). In addition, images of 14 mammal species and >20 other bird species were captured by the camera traps.

Based on the AIC values, the best fitting model used to compare the possible effect of camera placement (‘random’ *vs* ‘expert’) on taiga bean goose numbers captured by cameras had a zero-inflated negative Binomial error distribution. The camera placement did not have a significant effect on the number of taiga bean geese captured (p = 0.386).

Of the environmental variables, only mean temperature was retained in the best fitting GAM (p < 0.001). The best fitting model included the smooth of mean temperature, the factor variable province, two-way interaction of province and smooth of Julian day, two-way interaction of province and smooth of time period, three-way interaction province*Julian day*time period, random intercept of study site and random smooth of Julian day within year. This model explained nearly 70% of the variation in taiga bean goose activity (deviance explained of 69.5%, S1 Table).

**Fig 2:**
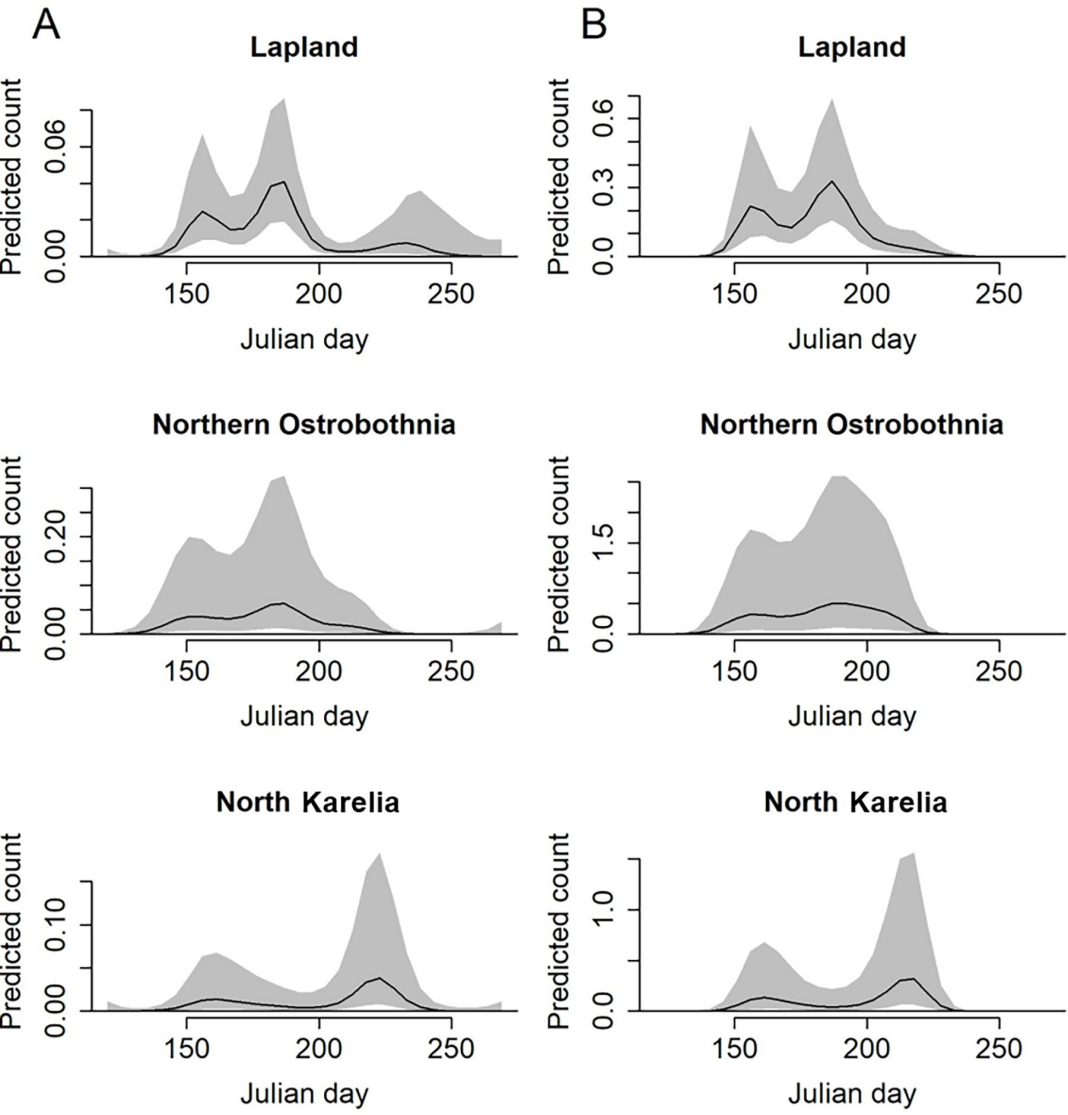
Seasonal variation in taiga bean goose count. Model predicted count (per unit effort) of taiga bean geese (mean with 95% prediction interval) with Julian day for the years a) 2018 and b) 2019.

**Fig 3:**
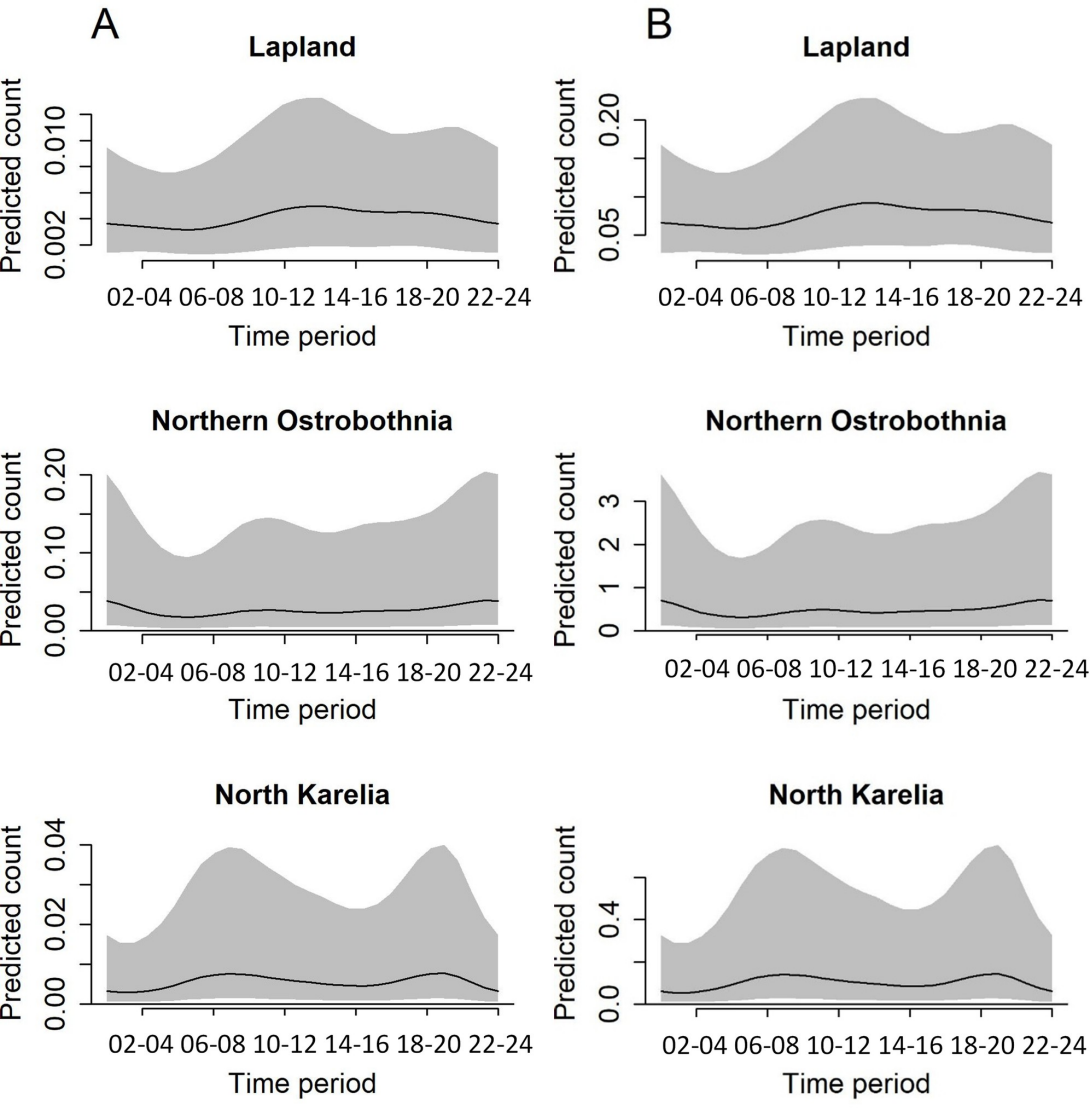
Diel variation in taiga bean goose count. Model predicted count (per unit effort) of taiga bean geese (mean with 95% prediction interval) with time period for the years a) 2018 and b) 2019.

**Fig 4:**
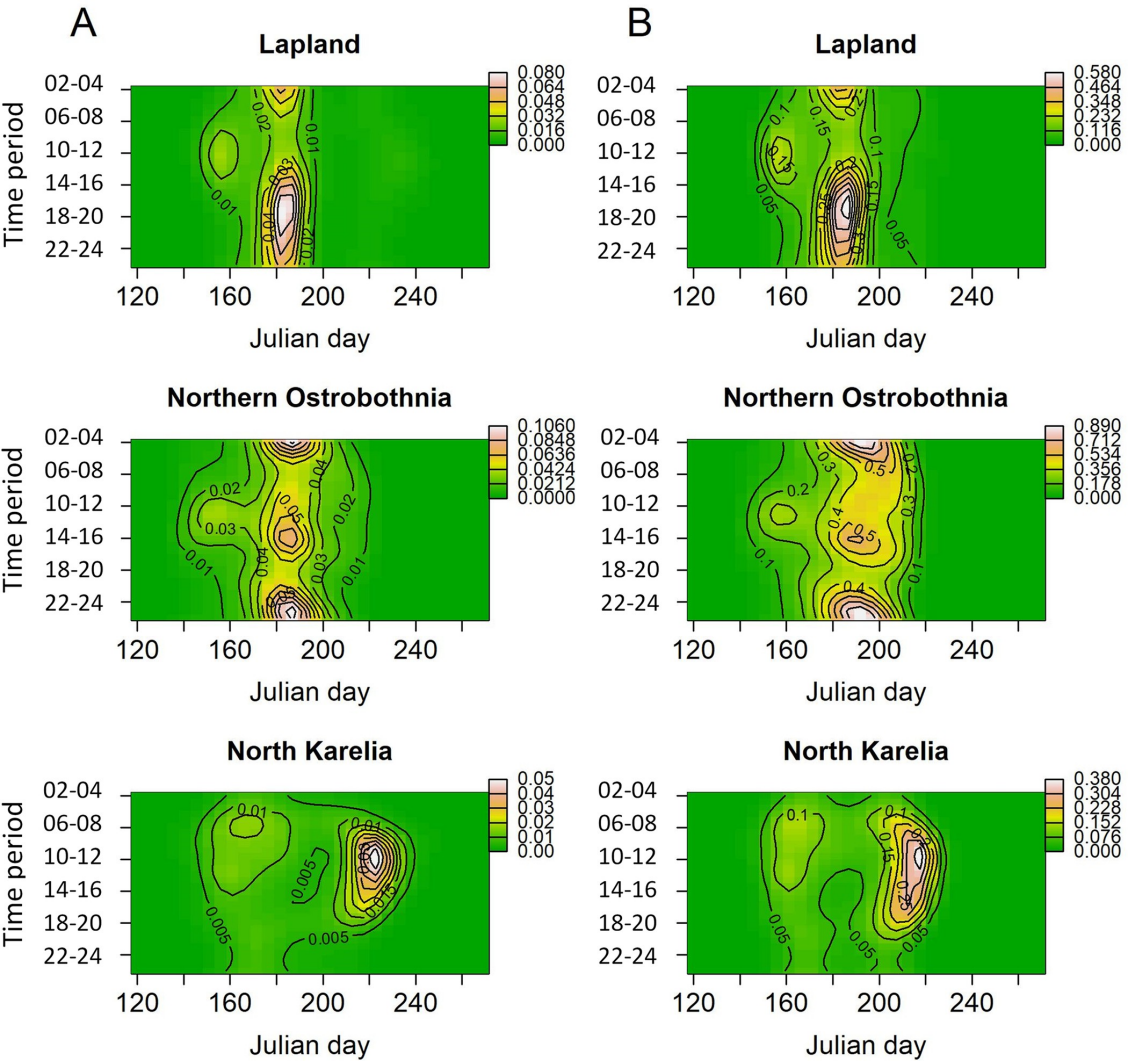
Combined seasonal and diel variation in taiga bean goose count. Model predicted mean count (per unit effort) of taiga bean geese with the interaction of Julian day and time period for the years a) 2018 and b) 2019. The uncertainty around the mean (95% CIs) is presented in S2 Fig.

Mean temperature had a slight positive effect on the number of taiga bean geese with larger counts predicted in warmer temperatures (S1 Fig). The model predicted count of taiga bean geese peaked on Julian day 185 and 186 (4th and 5th of July) in Lapland, on Julian day 185 and 189 (4th and 8th of July) in Northern Ostrobothnia and on Julian day 221 and 215 (9th and 3rd of August) in North Karelia, in years 2018 and 2019, respectively (Fig 2). Taiga bean goose activity peaked between 10:00 and 11:00 in Lapland in both years whereas the peak activity was predicted at around 00:00–02:00 in Northern Ostrobothnia and at 19:00 in North Karelia (Fig 3). Based on the predictions with the interaction of Julian day and time period (Fig 4), the taiga bean goose activity seemed to initially rise at around Julian day 145–150 (25th - 30th of May) in all provinces and years with the activity concentrated around the hours of midday and afternoon during this earlier rise. The timing of this spring activity was markedly different to the main July peak in Lapland and Northern Ostrobothnia, where the activity seemed to concentrate on night-time and around the hours of early morning with a decrease in daytime activity, especially in Lapland (Fig 4). Contrastingly, the activity seemed to peak during the daytime in North Karelia both in May and in August.

It is important to note that the random variation in the counts between the different peatlands in North Karelia and especially Northern Ostrobothnia, was large (with standard deviation of 1.371) resulting in large uncertainty (reflected in wide prediction intervals in Figs 2 and 3).

## Discussion

This study focused on testing the suitability of using remote camera traps in assessing temporal patterns in taiga bean goose activity at peatland ponds, estimated as counts of geese and accounting for the temporal and spatial variation in camera effort. We found no difference in counts between randomly placed cameras and cameras placed by experts. Further, we found that from the set of environmental variables tested, only temperature had a weak effect on bean goose activity with an increase in geese count with warmer mean temperatures. Overall, temporal variables (Julian day and time of day) explained most of the variation in goose activity, however, large random variation between the different peatlands and years led to large uncertainty around the predicted counts thus hindering our ability to draw definite conclusions on the activity patterns. Nevertheless, the results of this study give an indication of the best times to monitor bean goose in different areas of Finland, and possibly elsewhere in the breeding areas of the species, and may thus help to reduce the costs of monitoring. However, taiga bean goose activity patterns observed in this study may be very different in other parts of the vast distribution area of the subspecies. Moreover, the home ranges of breeding geese can extend over 10–20 km^2^ (and beyond), consisting of a complex of different mire forests, ponds, and small streams. In different stages of the breeding cycle taiga bean geese use these biotopes to a different degree, depending on the availability of preferred food, shelter, and on social relationships [16].

As hypothesized, temperature had a positive effect on taiga bean goose activity. However, wind speed or rain accumulation did not affect the count of taiga bean geese. This may be at least partly explained by the large variation in the prevailing temperature throughout the whole study period (-1.9–31.0°C in 2018 and -7.7–31.1°C in 2019, see S2 Table), i.e. the significant effect of temperature was likely linked to the increase in temperature from May to August. In contrast, variation in the average wind speed or rain accumulation was relatively small. Nevertheless, assuming that environmental conditions were typical in the study years, the results suggest that the probability of observing taiga bean geese in the breeding areas is not particularly dependent on wind and rain conditions.

There were differences between the regions in seasonal and diel activity patterns of the moulting taiga bean geese. In Lapland, the geese were observed most frequently between the 24th of June and 14th of July. During that period most of the geese were observed during the night and the hours of early morning, with a decline in activity between 12:00 and 20:00. Also in Northern Ostrobothnia, the peak started at the same time, but lasted longer (until 29th of July). In addition, the geese were most active over two long periods throughout the day: in 20:00–06:00 and in 08:00–16:00. In contrast, in the southernmost region, North Karelia, the largest counts occurred much later, between the 19th of July and the 13th of August, which may indicate more activities related to migration rather than moulting. In addition, the diel taiga bean goose activity in North Karelia was concentrated around the daytime hours (06:00 and 20:00) both in May and during the August peak without the seasonal switch in activity from daytime in May to being active mostly at night-time in July that was observed especially in Lapland. This further supports the hypothesis that the later peak in taiga bean goose activity in the lower latitude peatlands may be associated with a different type of behaviour compared to the geese observed in the higher latitude locations (moulting *vs* preparing for migration).

Overall, the underlying causes for the regional differences in diel activity patterns are likely to be varied but could be explained by, for example, differences in the timing of hatching of goslings. In waterfowl, including geese, the most critical time for growth is from the time of hatching up until they are about two weeks of age when their energy demand is the highest [29, 43]. The fact that at least in Northern Ostrobothnia, the activity of taiga bean geese increased to cover most of the day during the peak period in July could indicate increased demands to satisfy the energy requirements of goslings. On the other hand, the presence of visual predators, such as the golden eagle, *Aguila chrysaetos*, which is an important predator of taiga bean goose [44] and more abundant in northern Finland, could partly explain why the activity in Lapland was concentrated on the night time and early hours of morning.

Taiga bean geese nest and incubate their eggs in May with goslings hatching typically in June [5, 45]. There was substantial variation in the dates when first goslings started to appear in the photos, both between years and between regions. In 2018, the first goslings were counted on the 2nd and 3rd of June in Northern Ostrobothnia and North Karelia, respectively, whereas the first goslings did not appear in images from Lapland until the 29th of June (results not shown). Similarly, in 2019, the goslings were first observed in North Karelia on the 24th of May, the 28th of May in Northern Ostrobothnia but not until the 8th of June in Lapland. These results may indicate later hatching of taiga bean goose at higher latitudes due to later onset of spring. However, our study included data collected over only two breeding seasons and more research is thus needed to fully investigate the breeding phenology of taiga bean goose and the drivers affecting it.

In this study, we focused on quantifying the activity patterns, but camera traps have also other implications for monitoring. For example, due to taiga bean geese being potentially individually identifiable [46] based on neck bands or natural marks such as variation in bill pattern [47], regional abundance could possibly be estimated through capture-recapture methods. Moreover, camera traps are shown to increase the number of resightings of marked individuals at moulting sites [48]; indeed, a total of 12 different satellite tagged or colour marked taiga bean geese were observed in this study. These observations may add to the knowledge of the conservation status of the sub-population indicating whether the numbers are increasing or decreasing, at least on a local scale. Moreover, the likelihood of an individual occurring on a specific site or an area *and* being captured by camera traps could be quantified by combining the data from camera traps and from individuals simultaneously equipped with transmitters. This information could then be used for monitoring.

Camera traps may provide information not only on occupancy or abundance of geese, but also detailed data on varied demographic and biological aspects such as brood size, survival of goslings, predation, and site fidelity. In addition, camera trapping focusing on a single species can opportunistically capture data on other species that could be incorporated in further studies of the species’ ecology. In this study, a variety of bird and mammal species were recorded, such as potential predators (large and medium-sized mammals such as the wolverine, *Gulo gulo*, Eurasian lynx, *Lynx lynx*, and red fox, *Vulpes vulpes*) and competitors (whooper swan, *Cygnus cygnus*). Whooper swans are territorial, defending a nest site not only against conspecifics, but also showing aggression towards other species [49]. The increased whooper swan population is suggested to be one potential reason for the decline of the taiga bean goose population [11, 50], but this is mostly based on anecdotal evidence with long-term studies lacking. Future studies using camera traps could look into interactions and long-term co-existence of taiga bean goose and whooper swan.

Our results show that long-term monitoring with game camera traps provide a new potential unobtrusive approach for studying behavioural patterns of taiga bean goose and can increase knowledge on the ecology of this little-known subspecies. Body size and the behavioural patterns of the taiga bean goose are favourable for camera trap studies, as large ground-dwelling birds are shown to be captured more often by camera traps [51, 52]. Particularly the time window of our study, which was concentrated around the breeding season when adults are with goslings or flightless due to the moult, is optimal time for camera trapping. However, nesting success can affect regional abundances of geese; a telemetry study showed that taiga bean goose pairs that failed in nesting migrated from breeding areas in northern Finland to the Kola peninsula for moulting [53].

Manual analysis of the vast image data is very time-consuming and is thus one major weakness of camera trapping. With thousands of triggers expected within a month, this can result in over 5,000 images per memory card that need to be processed. Therefore, if the time windows for monitoring the size of the local population could be narrowed to e.g., one week based on the results of this and further studies, and perhaps also focused on certain times of the day, the total number of images per camera should be reduced to a more manageable amount. Another option would be to develop analytical tools (including software for automated image recognition, processing, and storage) that would likewise reduce the manual processing time of images. A further potential weakness of camera trapping when the individuals are not individually identified, is the movement of the animals which can lead to counting the same animal twice. However, we do not consider this as a major source of bias in this study as our goal was not to estimate absolute abundance of taiga bean geese but to quantify their activity patterns.

The global taiga bean goose counts are carried out in staging and wintering areas [54], but also regional breeding population estimates are urgently needed for sustainable harvest management purposes. Such regional estimates could be used to inform localized decision making on whether and where and when the hunting of the taiga bean goose should be allowed. In remote peatlands, the assessments by traditional ground counts are, however, logistically very challenging and time consuming to carry out. Therefore, helicopter-based surveys have been suggested as a potential monitoring method to supplement the ground counts, and they have been experimented in Finland during the moulting season over recent years [55]. In addition, alternative remote censusing methods, such as drone counts have been recently tested, but the results have not been promising, mainly due to the limited survey range of the unmanned aircraft and sporadic occurrence of the taiga bean geese. Moreover, disturbance effects by drones [17] and other aircrafts cannot be excluded. Therefore, non-invasive camera trapping could be one promising option for further development of alternative population estimation methods also for taiga bean goose [56]. In addition, the results of this study on activity patterns have direct implications for the current monitoring methods as they provide an indication of the most optimal seasonal and diurnal time windows to execute the censuses.

## Conclusions

We found camera traps to be an effective way to collect information on the elusive taiga bean goose. The activity of the geese varied both temporally and geographically between different regions of Finland, and these regional differences in the timing of the peak activity should be taken into account when planning the censuses. For example, there may be a need to focus the counts to the hours of early morning, especially in Lapland. It is also notable that randomly placed camera traps provided similar results compared to the ones placed by experts, which may indicate that the study site (peatland) is more important than the exact placement of the camera. This suggests that camera traps placed by non-expert citizens could be used for monitoring numbers of taiga bean geese in the breeding areas. Much of the variation in the taiga bean goose activity was explained by between site random variation, and it is possible that we have failed to incorporate some important ecological or environmental covariates, such as vegetation cover or type, in the model. Therefore, future studies should test a larger set of covariates, preferably after accumulating a dataset spanning multiple years, to fully resolve the breeding and moulting phenology of taiga bean goose and the factors affecting it. Examining moulting phenology in the context of long-term meteorological data would be especially important, as this sub-arctic species may be more vulnerable to the effects of the ongoing climate change.

## Supporting information

S1 Fig Taiga bean goose count with varying temperature. Model predicted count (per unit effort) of taiga bean geese (mean with 95% prediction interval) with varying temperature for the years a) 2018 and b) 2019.
(TIF)

S2 Fig Seasonal and diel variation in taiga bean goose count. Predicted mean count (black) of taiga bean geese with upper (red) and lower (green) 95% CIs, predicted with the interaction of Julian day and time period in the best fitting GAMM for the years a) 2018 and b) 2019. The 12 two-hour time periods (z-axis) correspond times from 00:00–02:00 to 22:00–00:00.
(TIF)

S1 Table Summary of candidate models ran to investigate the effects of temporal, spatial and environmental variables on taiga bean goose activity. (DOCX)

S2 Table Range of the variables used in the models for taiga bean goose activity. The environmental variables, recorded on an hourly basis, were extracted from altogether 33 weather stations located within 50 km radius from a specific peatland pond using a custom R-script (see Methods) and averaged over the two-hour time period.
(DOCX)

S1 Data Taiga bean goose dataset with environmental variables. (ZIP)
