# Supplementary figures and images for: Seasonal and diel activity patterns of the endangered taiga bean goose (Anser fabalis fabalis) during the breeding season, monitored with camera traps
Source: PLoS One. 2021 Jul 15;16(7):e0254254. doi: 10.1371/journal.pone.0254254 (PMC8282086; doi:10.1371/journal.pone.0254254)

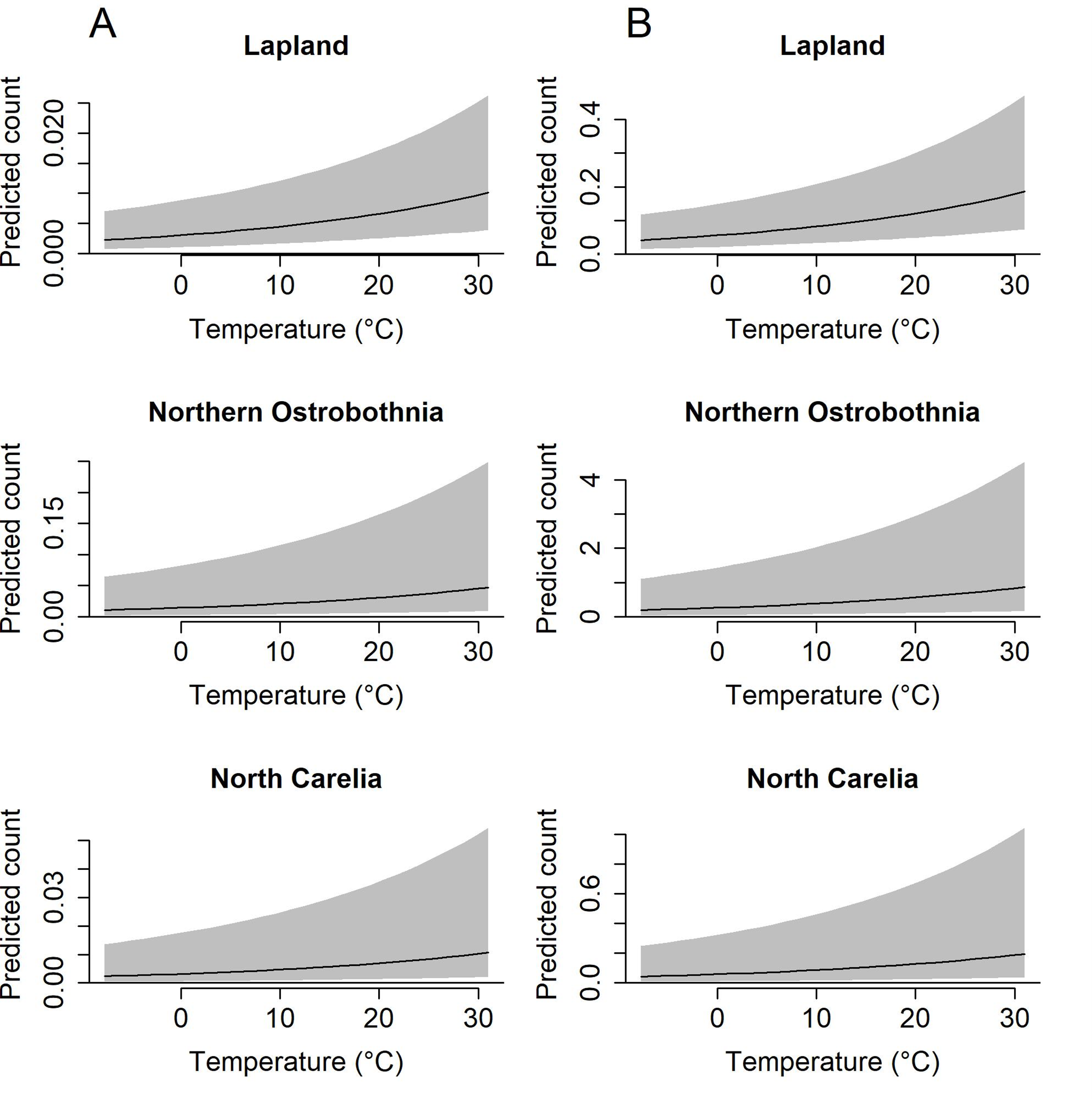

Supplement: S1 Fig — Model predicted count (per unit effort) of taiga bean geese (mean with 95% prediction interval) with varying temperature for the years a) 2018 and b) 2019. (TIF) [file pone.0254254.s001.tif]

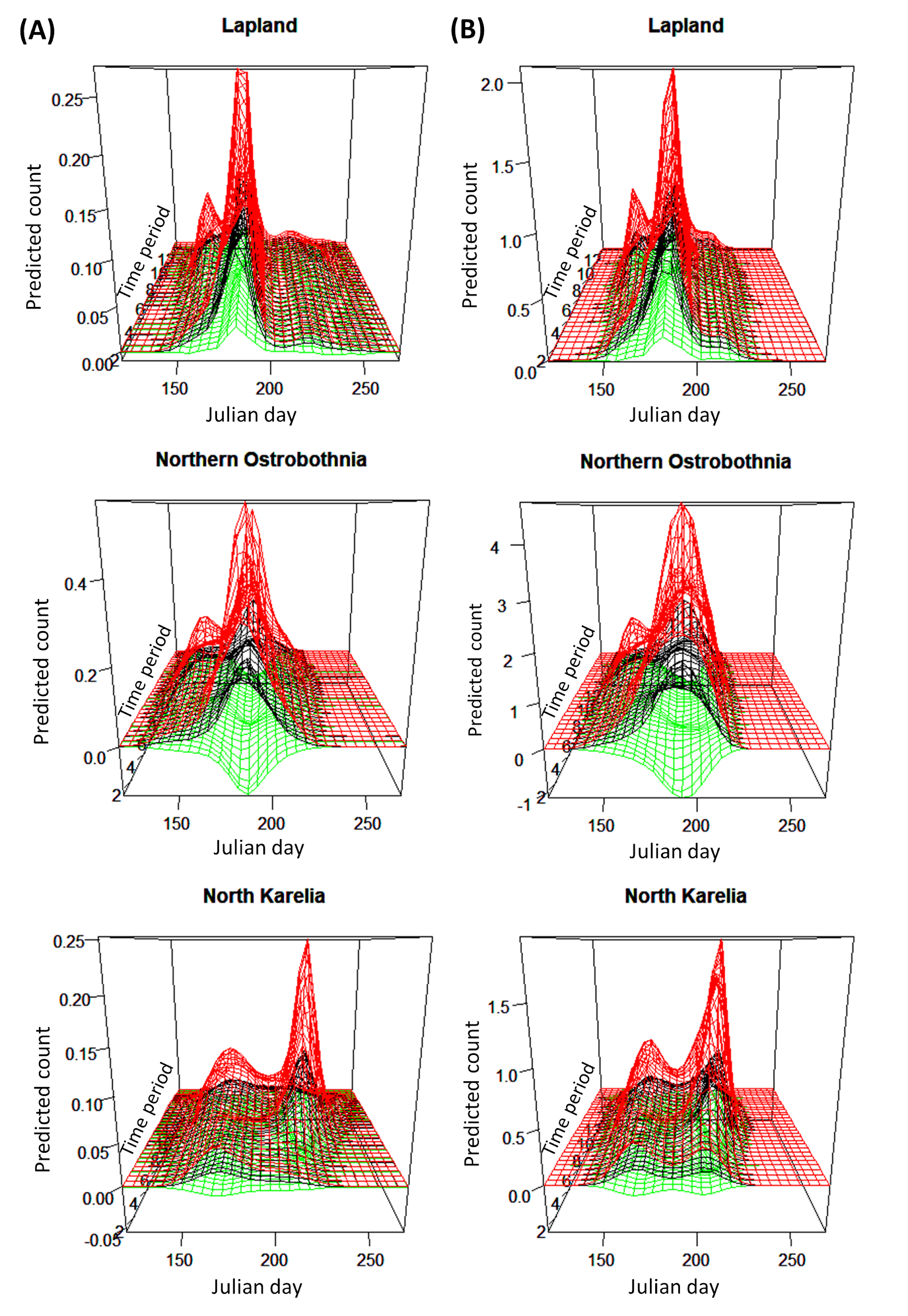

Supplement: S2 Fig — Predicted mean count (black) of taiga bean geese with upper (red) and lower (green) 95% CIs, predicted with the interaction of Julian day and time period in the best fitting GAMM for the years a) 2018 and b) 2019. The 12 two-hour time periods (z-axis) correspond times from 00:00–02:00 to 22:00–00:00. (TIF) [file pone.0254254.s002.tif]
