# Supplementary material for: Seasonal and diel activity patterns of the endangered taiga bean goose (Anser fabalis fabalis) during the breeding season, monitored with camera traps
Source: PLoS One. 2021 Jul 15;16(7):e0254254. doi: 10.1371/journal.pone.0254254 (PMC8282086; doi:10.1371/journal.pone.0254254)
Supplement: S1 Table — (DOCX) [file pone.0254254.s003.docx]

**S1 Table. Summary of candidate models ran to investigate the effects of temporal, spatial and environmental variables on taiga bean goose activity.**

| Model (without random variables) | Variables | | Smoothness selection method^a^ | degrees of freedom | AIC | Deviance explained (%) |
| --- | --- | --- | --- | --- | --- | --- |
|  | Fixed | Random |  |  |  |  |
| M_1_ | Julian day + time period | - | ML | 14.35 | 44158.00 | 48.7 |
| M_2_ | Province + Julian day + time period | - | ML | 16.39 | 44028.14 | 51.2 |
| M_3_ | Province + Julian day + time period + temperature | - | ML | 17.82 | 44019.06 | 51.5 |
| M_4_ | Province + Julian day + time period + temperature + rainfall | - | ML | 18.86 | 44020.26 | 51.5 |
| M_5_ | Province + Julian day + time period + temperature + rainfall + wind speed | - | ML | 20.02 | 44022.39 | 51.5 |
| M_6_ | Province + Julian day + time period + temperature + wind speed | - | ML | 18.87 | 44020.96 | 51.5 |
| M_7_ | Province + Julian day + time period + Julian day:time period + temperature ^b^ | - | ML | 39.55 | 44021.46 | 52.9 |
| M_8_ | Province + Julian day:province + time period:province + Julian day:time period:province + temperature ^b^ | - | ML | 75.20 | 43996.26 | 55.6 |
|  |  |  |  |  |  |  |
| Model (with random variables added) |  |  |  |  |  |  |
| M_8_ | Province + Julian day:province + time period:province + Julian day:time period:province + temperature ^b^ | - | REML | 73.87 | 43993.62 | 55.6 |
| M_8__ri | Province + Julian day:province + time period:province + Julian day:time period:province + temperature ^b^ | Random intercepts of year and site | REML | 99.54 | 43411.75 | 68.9 |
| M_8__rsl | Province + Julian day:province + time period:province + Julian day:time period:province + temperature ^b^ | Random slope of Julian day within a year, random intercept of site | REML | 100.32 | 43413.30 | 68.9 |
| M_8__rsl2 | Province + Julian day:province + time period:province + Julian day:time period:province + temperature ^b^ | Random slope of Julian day within a year fitted separately for each province, random intercept of site | REML | 102.57 | 43407.31 | 69.1 |
| M_8__risl | Province + Julian day:province + time period:province + Julian day:time period:province + temperature ^b^ | Random intercept and slope of Julian day within year, random intercept of site | REML | 99.53 | 43411.74 | 68.9 |
| M_8__rsm | Province + Julian day:province + time period:province + Julian day:time period:province + temperature ^b^ | Random smooth of Julian day within year, random intercept of site | REML | 104.74 | 43397.64 | 69.5 |

^a^ML = Maximum Likelihood, REML = Restricted Maximum Likelihood

^b^an interaction is denoted with a “:”
