# Supplementary material for: Seasonal and diel activity patterns of the endangered taiga bean goose (Anser fabalis fabalis) during the breeding season, monitored with camera traps
Source: PLoS One. 2021 Jul 15;16(7):e0254254. doi: 10.1371/journal.pone.0254254 (PMC8282086; doi:10.1371/journal.pone.0254254)
Supplement: S2 Table — The environmental variables, recorded on an hourly basis, were extracted from altogether 33 weather stations located within 50 km radius from a specific peatland pond using a custom R-script (see Methods) and averaged over the two-hour time period. (DOCX) [file pone.0254254.s004.docx]

**S2 Table.** **Range of the variables used in the models for taiga bean goose activity.** The environmental variables, recorded on an hourly basis, were extracted from altogether 33 weather stations located within 50 km radius from a specific peatland pond using a custom R-script (see Methods) and averaged over the two-hour time period.

| **Temporal/spatial variables** | **Province** | **Year** | |
| --- | --- | --- | --- |
|  |  | **2018** | **2019** |
| **Julian day** | Lapland | 144 - 241 | 120 - 269 |
|  | Northern Ostrobothnia | 127 - 230 | 120 - 269 |
|  | North Karelia | 136 - 252 | 120 - 269 |
| **Time period** | Lapland | 00-02 - 22-24 | 00-02 - 22-24 |
|  | Northern Ostrobothnia | 00-02 - 22-24 | 00-02 - 22-24 |
|  | North Karelia | 00-02 - 22-24 | 00-02 - 22-24 |
| **No. sites** | Lapland | 2 | 5 |
|  | Northern Ostrobothnia | 5 | 8 |
|  | North Karelia | 9 | 13 |

| **Environmental variables** | **Province** | **Year** | |
| --- | --- | --- | --- |
|  |  | **2018** | **2019** |
| **Mean temperature (°C)** | Lapland | 1.3 - 30.2 | -7.7 - 29.3 |
|  | Northern Ostrobothnia | -1.9 - 31 | -6.1 - 30.1 |
|  | North Karelia | -0.4 - 30.5 | -3.4 - 28.7 |
| **Mean rain accumulation (mm)** | Lapland | 0 - 9.2 | 0 - 6.5 |
|  | Northern Ostrobothnia | 0 - 3.7 | 0 - 13.7 |
|  | North Karelia | 0 - 2.5 | 0 - 5.0 |
| **Mean wind speed (m/s)** | Lapland | 0.5 - 11.2 | 0.2 - 10.4 |
|  | Northern Ostrobothnia | 0.3 - 10.0 | 0.3 - 11.8 |
|  | North Karelia | 0.0 - 7.1 | 0.0 - 6.6 |
